# Osteonecrosis of the femoral head in post-COVID-19 patients: a retrospective comparative study

**DOI:** 10.1186/s13018-025-05657-8

**Published:** 2025-04-10

**Authors:** Jichang Seong, Abduaziz Babakulov, Saodat Asilova, Babamukhamedova Shakhnoza, Makhmudova Nodira, Akbarjon Mirzayev

**Affiliations:** 1 School of Medicine, Central Asian University, Tashkent, 111221 Uzbekistan; 2 Department of Orthopedics and Traumatology, Akfa Medline University Hospital, Tashkent, 100211 Uzbekistan; 3 Department of Orthopedics and Traumatology, Kimyo University Hospital, Tashkent, 100121 Uzbekistan

**Keywords:** Avascular necrosis, COVID-19, Dexamethasone, Femoral head, Methylprednisolone, Osteonecrosis, Steroid, Vaccination

## Abstract

**Background:**

The COVID-19 pandemic has claimed many lives and continues to impact individuals through post-COVID-19 conditions. Osteonecrosis of the femoral head (ONFH) is increasingly recognized as a major post-COVID-19 complication, yet most studies are limited to case reports and small series. This study aimed to evaluate COVID-19-related factors potentially contributing to ONFH development in post-COVID-19 patients.

**Methods:**

A retrospective analysis was conducted on 84 patients with ONFH and a confirmed history of COVID-19. Baseline characteristics were collected, and patients were categorized into the following groups for comparative analysis: (1) vaccinated vs. unvaccinated, (2) unilateral vs. bilateral ONFH, (3) dexamethasone (DEX) and methylprednisolone (MPS) vs. DEX therapy, and (4) Association Research Circulation Osseus (ARCO) stage 2 vs. stage 3. Group differences and associations were analyzed.

**Results:**

The DEX and MPS-treated group had a greater extent of COVID-19 lung involvement compared to the DEX-treated group (60% vs. 30%, *p* = 0.002), as well as longer hospital stays in both general ward (14.2 days vs. 10.6 days, *p* = 0.018) and ICU (5.5 days vs. 2 days, *p* = 0.020). The DEX and MPS-treated group also had a longer duration of steroid therapy (19.3 days vs. 12.3 days, *p* < 0.001) and received higher DEX-equivalent cumulative steroid doses (380 mg vs. 125 mg, *p* < 0.001). Notably, ONFH symptoms developed earlier in the DEX and MPS-treated group compared to the DEX-treated group (7.5 months vs. 12 months, *p* = 0.004). Multivariable logistic regression analysis identified cumulative steroid dose as the sole predictor of ONFH severity (OR: 1.015, 95% CI: 1.001–1.028, *p* = 0.032), with ARCO stage 3 patients receiving higher cumulative steroid doses than stage 2 patients (240 mg vs. 126 mg, *p* = 0.018).

**Conclusions:**

Our study demonstrated that cumulative steroid dose is the primary determinant of ONFH severity in post-COVID-19 patients. Additionally, combined use of corticosteroids may accelerate the onset of ONFH, highlighting the need for cautious steroid management in COVID-19 patients.

## Background

In 2020, World Health Organization (WHO) declared Coronavirus disease 2019 (COVID-19) as a global pandemic [1]. Since its emergence, severe acute respiratory syndrome coronavirus-2 (SARS-CoV-2) has spread rapidly across the globe, infecting over 777 million people and causing more than 7 million deaths as of December 2024 [2, 3]. Moreover, the pandemic profoundly altered daily life, disrupting societal norms and significantly impacting educational training worldwide [4]. Over time, accumulating clinical data have highlighted the post-infectious complications of COVID-19, collectively termed as post-COVID-19 conditions [5]. These conditions impact nearly every organ system, including respiratory, cardiovascular, gastrointestinal, renal, and musculoskeletal systems [6, 7].

One such concerning complication is osteonecrosis (ON), particularly of the femoral head (FH) [7, 8]. ONFH is a debilitating condition caused by impaired blood flow to the proximal femur [9]. It primarily affects young adults in their 20s and 40s and may lead to severe hip pain, progressive arthritis, and eventual collapse of the femoral head if left untreated, necessitating total hip arthroplasty (THA) [9, 10]. However, given the limited lifespan of THA implants, young patients frequently require revision surgeries during their lifetime [10]. Therefore, clinicians must maintain a high index of suspicion when assessing hip and groin pain in patients with risk factors for ONFH, as early diagnosis before femoral head collapse allows for timely joint-preserving treatments, reducing morbidity and improving long-term outcomes [10, 11].

Corticosteroids are widely recognized as the leading cause of non-traumatic ONFH [11]. During the pandemic, corticosteroids were extensively used in active COVID-19 patients to improve survival rates and shorten hospitalization [12]. Previous studies from the severe acute respiratory syndrome (SARS) pandemic demonstrated a close association between corticosteroid use and ON [13]. A meta-analysis reported that 32% of the convalescent SARS patients developed ON after glucocorticoid therapy, with ONFH being the most common presentation [14]. Experts anticipate a similar trend among COVID-19 survivors [14, 15]. However, the precise pathophysiology of ONFH remains unclear and likely involves multiple factors beyond corticosteroid use [11]. While emerging studies have documented ONFH in post-COVID-19 patients, most available research is limited to case reports and small case series [16, 17]. Therefore, this study aimed to comprehensively explore the potential association between COVID-19-related factors and ONFH.

## Methods

### Study design

This single-center retrospective study aimed to explore potential links between COVID-19-related factors and the development of ONFH in post-COVID-19 patients.

### Patient selection

Between January 2021 and December 2023, 556 ONFH patients were assessed and treated at Akfa Medline University Hospital. Of these, 84 patients meeting the specific inclusion and exclusion criteria were included in this study. The inclusion criteria required a confirmed prior history of COVID-19, managed either as an inpatient or outpatient, followed by a subsequent diagnosis of ONFH. Patients were excluded from the study if they had no or unidentifiable history of COVID-19, ONFH predating COVID-19 infection, or a history of alcohol consumption exceeding 400 ml/week. Additional exclusion criteria included patients with autoimmune diseases, chronic steroid treatment, oncological conditions or chemotherapy, and prior pelvic or femoral trauma or surgery.

### Study variables

Comprehensive patient data were collected, including demographic details (age and gender), anthropometric measurement (body mass index, BMI), and past medical history. COVID-19-related information included vaccination status, extent of COVID-19 lung involvement, hospitalization duration in general ward and intensive care unit (ICU), and details of steroid therapy (regimen type, cumulative dose, and duration). ONFH-specific data included symptom onset, severity, and affected joints. For comparative analysis, patients were categorized into the following groups: (1) vaccinated vs. unvaccinated, (2) unilateral vs. bilateral ONFH, (3) dexamethasone (DEX) and methylprednisolone (MPS) vs. DEX therapy, and (4) Association Research Circulation Osseus (ARCO) stage 2 vs. stage 3. Group differences and associations were analyzed accordingly.

### COVID-19 assessment

COVID-19 infection was confirmed using either a COVID-19 rapid antigen test or real-time polymerase chain reaction (RT-PCR). The extent of COVID-19 lung involvement was assessed based on prior chest computed tomography (CT) scans, with both lungs collectively considered as 100% for calculating the extent of involvement.

### ONFH assessment

The onset of ONFH symptoms, including groin pain, limited range of motion, and limping was documented. Diagnosis of ONFH was confirmed through radiographic evaluations using plain radiography, CT scans, or magnetic resonance imaging (MRI), either individually or in combination. ONFH severity was classified according to the ARCO staging system [18]. Imaging studies were conducted only after symptom onset, and ARCO staging was based on the most recent scans. Due to the small sample size of stage 1 patients (*N* = 2), these cases were excluded from statistical analysis. For simplicity, early 3A and late (3B) subdivisions of stage 3 were combined and collectively reported as stage 3.

### Steroid dose conversion

Cumulative steroid doses were expressed in DEX-equivalents. Since 6 mg of DEX is equivalent to 32 mg of MPS [19], MPS doses were converted using the following formula:

1 mg of MPS = 0.1875 mg of DEX [14].

### Statistical analysis

Data normality was assessed using the Shapiro-Wilk test and visually through Q-Q plots. Continuous variables were summarized as mean ± standard deviation (SD) for normally distributed data and as median with interquartile range (IQR) for non-normally distributed data. Categorical variables were presented as frequencies and percentages. Total percentages may exceed 100% due to rounding to one decimal place. For group comparisons, Mann-Whitney test was used for non-normally distributed data, while Student’s t-test was applied for normally distributed data. Chi-square test or Fisher’s exact test was used for categorical variables. When Chi-square test or Fisher’s exact test was statistically significant, a multivariable logistic regression analysis was performed. Effect sizes are reported as rank-biserial correlation (*r*_*B*_) for Mann-Whitney test, Cohen’s d (*d*) for Student’s t-test, and odd ratio (OD) for Chi-square test, Fisher’s exact test, and logistic regression. All statistical analyses were conducted using JASP version 0.19.2.0 (University of Amsterdam, Netherlands). A *p*-value less than 0.05 was considered statistically significant.

## Results

### Baseline characteristics

The baseline characteristics of the patients are summarized in Table 1. Patient ages ranged from 24 to 73 years, with a median age of 39.5 years (IQR 35-48.5 years). The majority of the patients fell within the 35–44 age group (*N* = 36, 42.9%). Males were predominant (*N* = 61, 72.6%), with a male-to-female ratio of 2.7:1. BMI ranged from 18.4 to 39.2 kg/m^2^, with a median of 24.8 kg/m^2^ (IQR 21.6–28.8 kg/m^2^). Many of the patients were within the normal weight range (*N* = 41, 48.8%). Comorbidities included diabetes mellitus (*N* = 4, 4.8%) and hypertension (*N* = 9, 10.7%).

Regarding COVID-19 vaccination, the majority were unvaccinated (*N* = 54, 64.3%). COVID-19 lung involvement was observed in 63 patients (75%), with an average lung involvement of 40.6 ± 24.1% (range 5–85%). Most patients required hospitalization in the general ward (*N* = 60, 71.4%), with a mean hospital stay of 11.4 ± 4.7 days (range 2–26 days). A minority required ICU admission (*N* = 20, 23.8%), with a mean ICU stay of 4.2 ± 2.3 days (range 4–24 days).

The cumulative steroid dose ranged from 4 to 446 mg, with a median of 160 mg (IQR 64.3-263.8 mg), and a mean treatment duration of 13.4 ± 6.7 days (range 1–30 days). The majority of the patients received DEX alone (*N* = 66, 78.6%), while 12 patients (14.3%) received both DEX and MPS.

In terms of ONFH, most patients were in ARCO stage 2 (*N* = 57, 67.9%), and bilateral FH involvement was observed in 50 patients (59.5%). Symptom onset ranged from 4 to 24 months, with a median onset of 12 months (IQR 8–15 months) (Table 1).

**Table 1:** The baseline characteristics of the patients

Variables	*N* (%)	Mean ± SD	Median (IQR)	Min-Max
**Age (Year)**		42.5 ± 10.5	39.5 (35-48.5)	24–73
< 25	1 (1.2)			
25–34	18 (21.4)			
35–44	36 (42.9)			
45–54	14 (16.7)			
55–64	13 (15.5)			
> 65	2 (2.4)			
**Gender**				
Male	61 (72.6)			
Female	23 (27.4)			
**BMI (kg/m** ^**2**^ **)**		25.1 ± 4.4	24.8 (21.6–28.8)	18.4–39.2
Underweight (< 18.5)	1 (1.2)			
Normal (18.5–24.9)	41 (48.8)			
Overweight (25-29.9)	31 (36.9)			
Obesity I (30-34.9)	9 (10.7)			
Obesity II (35-39.9)	2 (2.4)			
**Medical history**				
Alcohol intake (< 400 ml/week)	2 (2.4)			
Diabetes Mellitus	4 (4.8)			
Hypertension	9 (10.7)			
**COVID-19**				
Vaccination status				
Vaccinated	30 (35.7)			
Unvaccinated	54 (64.3)			
Lung involvement (%)		40.6 ± 24.1	40 (22.5–60)	5–85
Involved	63 (75)			
Uninvolved	21 (25)			
Hospitalization (days)				
General ward		11.4 ± 4.7	12 (8–14)	2–26
Yes	60 (71.4)			
No	24 (28.6)			
ICU		4.2 ± 2.3	4 (2–6)	1–8
Yes	20 (23.8)			
No	64 (76.2)			
**Steroid**				
Treatment duration (days)		13.4 ± 6.7	15 (8–18)	1–30
Treated	78 (92.9)			
Untreated	6 (7.1)			
Regimen				
DEX	66 (78.6)			
DEX + MPS	12 (14.3)			
Cumulative dose (mg)		173 ± 125	160 (64.3-263.8)	4-446
**ONFH**				
Symptom onset (months)		12 ± 5.3	12 (8–15)	4–24
ARCO stage				
Stage 1	2 (2.4)			
Stage 2	57 (67.9)			
Stage 3	25 (29.8)			
Joints involvement				
Unilateral	34 (40.5)			
Bilateral	50 (59.5)			

**Abbreviations**: Association research circulation osseus, ARCO; Body mass index, BMI; Coronavirus disease 2019, COVID-19; Dexamethasone, DEX; Intensive care unit, ICU; Interquartile range, IQR; Methylprednisolone, MPS; Osteonecrosis of femoral head, ONFH; Standard deviation, SD

### Vaccination status

We first examined the potential differences between vaccinated and unvaccinated patients. As summarized in Table 2, there were no significant differences between the two groups in terms of age, gender, and BMI. Both groups exhibited similar degrees of COVID-19 lung involvement and comparable hospitalization durations in both the general ward and ICU. Additionally, no significant differences were observed in steroid treatment duration, regimen type, or cumulative dose. The onset of ONFH symptoms, as well as ARCO staging and joint involvement, showed no significant variation between vaccinated and unvaccinated patients (Table 2). These results indicate that vaccination does not influence the development of ONFH.

**Table 2:** Comparative analyses between vaccination status and other variables

Variables	Vaccination status							*p*-value
	Vaccinated				Unvaccinated			
	*N* (%)	Mean ± SD	Median (IQR)		*N* (%)	Mean ± SD	Median (IQR)	
**Age (Year)**	30		38.5 (35-50.3)		54		41 (35.5–46.5)	0.667
**Gender**								0.258
Male	24 (80)				37 (68.5)			
Female	6 (20)				17 (31.5)			
**BMI (kg/m** ^**2**^ **)**	30		25.4 (22.2–27.9)		54		24.4 (21.5–29)	0.948
**COVID-19**								
Lung involvement (%)	24	38.8 **±** 19.5	35 (25–50)		39	41.8 **±** 26.7	40 (17.5–60)	0.804
Hospitalization (days)								
General ward	23	11.9 **±** 3.9			37	11 **±** 5.1		0.500
ICU	9	3.9 **±** 1.8			11	4.5 **±** 2.7		0.603
**Steroid**								
Treatment duration (days)	29	13.6 **±** 6.5	15 (8–18)		49	13.2 **±** 6.9	14 (8–18)	0.791
Regimen								0.727
DEX	24 (80)				42 (77.8)			
DEX + MPS	5 (16.7)				7 (13)			
Cumulative dose (mg)	29		170 (65–280)		49		160 (64–240)	0.717
**ONFH**								
Symptom onset (months)	30		12 (8–12)		54		11 (8–16)	0.552
ARCO stage								0.213
Stage 2	17 (56.7)				40 (74.1)			
Stage 3	11 (36.7)				14 (25.9)			
Joint involvement								0.074
Unilateral	16 (53.3)				18 (33.3)			
Bilateral	14 (46.7)				36 (66.7)			

**Abbreviations**: Association research circulation osseus, ARCO; Body mass index, BMI; Coronavirus disease 2019, COVID-19; Dexamethasone, DEX; Intensive care unit, ICU; Interquartile range, IQR; Methylprednisolone, MPS; Osteonecrosis of femoral head, ONFH; Standard deviation, SD

### Joint involvement

We next examined potential differences between unilateral and bilateral ONFH groups. As summarized in Table 3, there were no significant differences between the two groups in terms of age, gender, or BMI. COVID-19 lung involvement and hospitalization duration, in both general ward and ICU, were comparable between the two groups. Additionally, no significant differences were observed in steroid treatment duration, regimen type, or cumulative dose. The onset of ONFH symptoms and ARCO stage also showed no variation between unilateral and bilateral ONFH groups (Table 3). These results suggest that ONFH laterality is not influenced by the patients’ demographic factors or COVID-19-related factors.

**Table 3:** Comparative analyses between joint involvement and other variables

Variables	Joint involvement							*p*-value
	Unilateral				Bilateral			
	*N* (%)	Mean ± SD	Median (IQR)		*N* (%)	Mean ± SD	Median (IQR)	
**Age**	34		41.5 (36.3–52.5)		50		38 (34.3–45)	0.152
**Gender**								0.399
Male	23 (67.6)				38 (76)			
Female	11 (32.4)				12 (24)			
**BMI**	34	24.3 **±** 4.2	24 (21-27.2)		50	25.7 **±** 4.5	25.4 (22–29)	0.156
**COVID-19**								
Lung involvement (%)	26	45.2 **±** 24.3	42.5 (26.3-65)		37	37.4 **±** 23.8	30 (20-60)	0.210
Hospitalization (days)								
General ward	26	11.5 **±** 5.2			34	11.2 **±** 4.3		0.786
ICU	9	3.9 **±** 2.4			11	4.5 **±** 2.3		0.603
**Steroid**								
Treatment duration (days)	31	14 **±** 6.7			47	13 **±** 6.7		0.525
Regimen								0.153
DEX	24 (70.6)				42 (84)			
DEX + MPS	7 (20.6)				5 (10)			
Cumulative dose (mg)	31		200 (80-308.5)		47		138 (64–220)	0.156
**ONFH**								
Symptom onset (months)	34		12 (8–16)		50		12 (8-13.5)	0.733
ARCO stage								0.200
Stage 2	21 (61.8)				36 (72)			
Stage 3	13 (38.2)				12 (24)			

**Abbreviations**: Association research circulation osseus, ARCO; Body mass index, BMI; Coronavirus disease 2019, COVID-19; Dexamethasone, DEX; Intensive care unit, ICU; Interquartile range, IQR; Methylprednisolone, MPS; Osteonecrosis of femoral head, ONFH; Standard deviation, SD

### Types of steroid regimen

We further examined whether the type of steroid regimen influenced ONFH development. The results are summarized in Table 4. There were no significant differences in age, gender, or BMI between the two groups. However, patients treated with DEX and MPS had significantly greater COVID-19 lung involvement compared to those treated with DEX alone (60% vs. 30%, *p* = 0.002, r_B_ = 0.565). The DEX and MPS-treated group also had significantly longer hospitalization duration in both the general ward (14.2 days vs. 10.6 days, *p* = 0.018, *d* = 0.788) and ICU (5.5 days vs. 2 days, *p* = 0.020, r_B_ = 0.620). Additionally, the DEX and MPS-treated group had a significantly longer treatment duration (19.3 days vs. 12.3 days, *p* < 0.001, *d* = 1.136) and received a significantly higher cumulative dose (380 mg vs. 125 mg, *p* < 0.001, *r*_B_ = 0.957) than the DEX-treated group. Interestingly, ONFH symptoms developed significantly earlier in the DEX and MPS-treated group compared to the DEX-treated group (7.5 months vs. 12 months, *p* = 0.004, *r*_B_ = 0.523). Moreover, a significant association was observed between the steroid regimen type and ARCO stage (OR: 3.578, 95% CI: 1.004–12.745, *p* = 0.041) (Table 4). However, after adjusting for comorbidities (hypertension and diabetes mellitus), hospitalization (general ward and ICU), COVID-19 lung involvement, cumulative steroid dose, and treatment duration in a multivariable logistic regression model, the steroid regimen type was no longer independently associated with ARCO stage (OR: 1.047, 95% CI: 0.120–9.151, *p* = 0.967). Instead, cumulative steroid dose emerged as a significant predictor of ARCO stage (OR: 1.015, 95% CI: 1.001–1.028, *p* = 0.032). These results suggest that ONFH severity, as reflected by ARCO stage, is primarily determined by the cumulative steroid dose rather than the specific steroid regimen. Additionally, the earlier onset of ONFH symptoms in DEX and MPS-treated group may suggest a potential pharmacological synergism between the two corticosteroids.

**Table 4:** Comparative analyses between steroid type and other variables

Variables	Steroid regimen							*p*-value
	DEX				DEX + MPS			
	*N* (%)	Mean ± SD	Median (IQR)		*N* (%)	Mean ± SD	Median (IQR)	
**Age**	66		38 (35-49.5)		12		40 (32.3–46)	0.917
**Gender**								0.211
Male	50 (75.8)				7 (58.3)			
Female	16 (24.2)				5 (41.7)			
**BMI**	66		24.7 (22-28.9)		12		25.4 (23.7–28.3)	0.662
**COVID-19**								
Lung involvement (%)	51	36.3 **±** 23.3	30 (20-50)		12	59.2 **±** 18.3	60 (43.8-76.3)	0.002
Hospitalization (days)								
General ward	48	10.6 **±** 4.7			12	14.2 **±** 3.5		0.018
ICU	10	3 **±** 1.9	2 (2-4.5)		10	5.4 **±** 2.1	5.5 (4-7)	0.020
**Steroid**								
Treatment duration (days)	66	12.3 **±** 6.5			12	19.3 **±** 3.8		< 0.001
Cumulative dose (mg)	66		125 (61–200)		12		380 (360–388)	< 0.001
**ONFH**								
Symptom onset (months)	66		12 (8–15)		12		7.5 (5.8–9.3)	0.004
ARCO stage								0.041
Stage 2	46 (69.7)				5 (41.7)			
Stage 3	18 (27.3)				7 (58.3)			

**Abbreviations**: Association research circulation osseus, ARCO; Body mass index, BMI; Coronavirus disease 2019, COVID-19; Dexamethasone, DEX; Intensive care unit, ICU; Interquartile range, IQR; Methylprednisolone, MPS; Osteonecrosis of femoral head, ONFH; Standard deviation, SD

### ARCO stage

Lastly, we examined potential differences between ARCO stage 2 and 3 groups, with the results summarized in Table 5. Our analysis showed no significant differences in age, gender, or BMI between the two groups. The COVID-19 lung involvement and hospitalization duration (both general ward and ICU) were also comparable. Additionally, there were no significant differences in steroid treatment duration or the onset of ONFH symptoms between ARCO stage 2 and stage 3 groups. However, patients in ARCO stage 3 had received a significantly higher cumulative steroid dose compared to those in ARCO stage 2 (240 mg vs. 126 mg, *p* = 0.018, *r*_B_ = 0.337) (Table 5). These findings, consistent with our earlier results, further support the critical role of cumulative steroid dose in ONFH progression and severity.

**Table 5:** Comparative analyses between ARCO stage and other variables

Variables	ARCO stages							*p*-value
	Stage 2				Stage 3			
	*N* (%)	Mean ± SD	Median (IQR)		*N* (%)	Mean ± SD	Median (IQR)	
**Age**	57		42 (35–53)		25		38 (34–41)	0.104
**Gender**								0.702
Male	41 (71.9)				19 (76)			
Female	16 (28.1)				6 (24)			
**BMI**	57	25.3 **±** 4.5	25.4 (22–28)		25	24.8 **±** 4.4	24.2 (21–29)	0.600
**COVID-19**								
Lung involvement (%)	43	39.3 **±** 24.1	35 (22.5-55)		19	42.4 **±** 24.5	40 (22.5-55)	0.657
Hospitalization (days)								
General ward	38	11.3 **±** 4.8			21	11.5 **±** 4.6		0.856
ICU	11	4.3 **±** 2.2	5 (2.5-6)		9	4.1 **±** 2.6	3 (2-6)	0.939
**Steroid**								
Treatment duration (days)	51	12.9 **±** 6.8			25	15 **±** 6.1		0.205
Cumulative dose (mg)	51		126 (64–221)		25		240 (138–365)	0.018
**ONFH**								
Symptom onset (months)	57		12 (8–14)		25		12 (8–16)	0.947

**Abbreviations**: Association research circulation osseus, ARCO; Body mass index, BMI; Coronavirus disease 2019, COVID-19; Dexamethasone, DEX; Intensive care unit, ICU; Interquartile range, IQR; Methylprednisolone, MPS; Osteonecrosis of femoral head, ONFH; Standard deviation, SD

## Discussion

A recent retrospective cohort study demonstrated that a history of COVID-19 diagnosis was linked to a higher incidence of idiopathic ON in patients undergoing THA, suggesting that COVID-19 infection may contribute to the onset of ON [20]. Additionally, impaired mobility and prolonged hospitalization during COVID-19 may increase the risk of fragility fractures in the elderly, potentially predisposing them to ONFH [21]. While corticosteroid therapy during COVID-19 is considered the primary driver of ONFH [22], its pathophysiology remains poorly understood and is thought to involve multiple factors beyond corticosteroid use [11]. Therefore, our study aimed to identify potential COVID-19-related factors associated with ONFH development.

We first explored the impact of COVID-19 vaccination on ONFH. Our findings showed no significant differences between vaccinated and unvaccinated patients, suggesting that vaccination does not play a role in ONFH development. However, a recent case report described avascular necrosis (AVN) of the humeral head following the second dose of COVID-19 vaccine, suggesting a potential association between COVID-19 vaccination and AVN [23]. These discrepancies may stem from our study limitations, as vaccination status was self-reported and lacked details regarding the type and dose of vaccines administered. Additionally, patients’ habitual intake of vitamins C and D, both of which have been shown to improve oxygenation, reduce mortality, and decrease thrombosis risk in COVID-19 patients, may have also influenced our result [24]. Furthermore, as human leukocyte antigen (HLA) polymorphisms have been implicated in susceptibility, progression, and severity of SARS-CoV-2 infection, genetic predisposition may also contribute to post-COVID-19 ONFH, irrespective of vaccination status [25].

Next, we examined the differences between unilateral and bilateral ONFH groups. Although we found no significant differences between the two groups, we observed that the majority of our patients (59.5%) presented with bilateral ONFH. Similarly, Sehrawat et al. reported that 78% of their study population had bilateral hip AVN [26]. Several other studies have also documented a surprisingly high incidence of bilateral AVN following COVID-19 infection [17, 22, 27]. Hogea et al. observed that 34.6% of their COVID-19-positive patients presented with bilateral ONFH, and suggested that the systemic effects of the virus on vascular homeostasis and bone metabolism could contribute to this phenomenon [8]. Indeed, SARS-CoV-2 is known to directly infect vascular endothelial cells via angiotensin converting enzyme 2 (ACE2) receptor, leading to endothelial dysfunction and vascular integrity disruption [28–30]. The resulting endothelial damage exposes the basement membrane and extracellular matrix collagen to circulating platelets, triggering platelet aggregation and thrombosis [28, 31]. Additionally, inflammatory cytokines such as interleukin (IL)-1β and tumor necrosis factor (TNF)-α activate endothelial cells, promoting expression of surface proteins such as P-selectin, von Willebrand factor (vWF), and fibrinogen, which facilitate platelet and leukocyte recruitment [28, 30, 32]. Moreover, the severe immune response induced by the virus activates the coagulation cascade, predisposing individuals to a hypercoagulable state [12]. Since ACE2 receptors are also expressed in bone marrow-derived stem/progenitor cells, SARS-CoV-2 may target these cells, leading to impaired skeletal repair and accelerated bone loss [12]. Overall, COVID-19-associated vascular changes, including endothelial inflammation, vascular narrowing, thrombotic microangiopathy, capillary dysfunction, and impaired tissue oxygenation, may collectively increase the risk of ON [12, 33]. We noted that regardless of unilateral or bilateral ONFH, our patients received far less prednisolone (PS)-equivalent cumulative doses of corticosteroids that need to meet the ARCO criteria for corticosteroid-associated ONFH [18]. Therefore, we support the hypothesis that the systemic effects of SARS-CoV-2 on vascular homeostasis may play a role in the development of ONFH.

We further examined the differences between the patients treated with DEX and MPS and DEX alone. Notably, we found out that DEX and MPS-treated group experienced an earlier ONFH symptoms compared to the DEX-treated group. We hypothesize that this may be due to the pharmacological synergism between the two corticosteroids, which could amplify both their therapeutic and adverse effects. Evidence for positive synergism has been documented in various settings. For instance, a study demonstrated that the combination of PS and low-dose DEX dramatically enhanced the anti-leukemic effect of PS in childhood lymphoblastic leukemia compared to PS alone [34]. Similarly, a retrospective study found that combined DEX and PS therapy reduced the severity of status asthmaticus exacerbation compared to PS alone [35]. Additionally, the combination of DEX and MP was shown to mitigate severe pulmonary inflammation caused by paraquat poisoning [36]. In the context of COVID-19, a case report described the use of combined DEX and MPS, with the ONFH symptoms developing 56 days after recovery [37]. Another study compared moderate COVID-19 patients treated with DEX alone to severe cases treated with a combination of DEX and MPS. Although ONFH symptoms developed similarly in both groups, the study did not include a statistical analysis [38]. These controversial findings underscore the need for further investigation into the synergistic adverse effects of combined corticosteroid therapy, as well as the individual effect of each steroid in the development of ONFH.

Initially, our findings indicated that ONFH severity was strongly associated with the type of steroid regimen. Specifically, we observed that patients in the DEX and MPS-treated group exhibited a greater extent of COVID-19 lung involvement, had longer stays in both the general ward and ICU, and received longer and higher cumulative doses of steroids compared to those treated with DEX alone. Teeratakulpisarn et al. reported that greater cumulative doses of DEX-equivalents were linked to prolonged hospital and ICU stays [39], and Dolat Abadi et al. demonstrated that higher doses and extended duration of steroid therapy in COVID-19 patients are linked to a greater risk of developing ONFH [40]. However, further multivariable logistic regression analysis showed that the cumulative steroid dose is the sole predictor of ONFH severity. This result was further supported by a subsequent comparative analysis between the two ARCO stages, where cumulative steroid dose was the only variable significantly different between ARCO stage 2 and stage 3 groups. In line with our results, a study reported that severe COVID-19 patients treated with a combination of DEX and MPS exhibited greater ONFH severity compared to the moderate cases treated with DEX alone, and that rise in ONFH incidence during COVID-19 pandemic is attributed to the use of high-dose corticosteroid therapy in patients hospitalized with severe COVID-19 pneumonia [38]. These results suggest that factors such as greater extent of COVID-19 lung involvement, prolonged hospital stays, and steroid treatment durations are merely indirect reflections of higher cumulative steroid doses.

Several treatment modalities have been explored for the management of ONFH. The initial treatment typically involves conservative approaches, including physiotherapies such as extracorporeal shockwaves, pulsed electromagnetic fields, and hyperbaric oxygen therapy, as well as medications such as bisphosphonates or non-steroidal anti-inflammatory drugs (NSAIDs) [41, 42]. If the conservative management fails, surgical procedures such as core decompression, bone grafting (with or without mesenchymal stem cell injection), and rotational osteotomy are indicated for early to moderate ONFH, while THA is reserved as a last resort for end-stage disease [41, 42]. Sadile et al. reported that core decompression is not superior to other joint-preserving strategies and may be less successful in management of ONFH [43]. However, a recent meta-analysis by Migliorini et al. found that combining core decompression with bone marrow-derived cell therapy reduced pain and lowered the rate of THA compared to core decompression alone [44]. On the other hand, Quaranta et al. demonstrated that about one-third of osteotomies performed in ONFH patients were eventually converted to total hip replacement (THR) within seven years, suggesting that THR may be the preferred primary option for elderly patients [42]. Additionally, a recent study identified male gender, prolonged symptom duration, higher visual analog scale (VAS) scores, and lower Harris hip score (HHS) scores as negative prognostic factors following ONFH treatment [45]. In young patients with skeletal immaturity, treatment strategies remain controversial due to limited evidence and unpredictable outcomes [41]. Therefore, future research is warranted to establish standardized treatment protocols, optimize patient selection for different interventions, and improve long-term outcomes in ONFH management.

We acknowledge that several limitations are present in our study. First, as a single-center study, our findings may not be generalizable to the broader population. Second, we did not assess the impact of comorbidities such as diabetes mellitus and hypertension on the development of ONFH due to the relatively small sample size. Third, the vaccination status of our patients was self-reported, lacking detailed information regarding the type, dose, and timing of vaccines administered. Additionally, recall bias among patients cannot be ruled out as a potential factor influencing our results. A future study with more precise, comprehensive, and verified data is necessary to clarify the potential link between COVID-19 vaccination and ONFH. Fourth, we used standard imaging modalities, including plain radiography, CT, and MRI, to assess the ONFH severity. However, these imaging modalities may not detect early subclinical ONFH cases. Incorporating advanced imaging techniques such as perfusion MRI and dynamic contrast-enhanced MRI could provide further insights. Fifth, we could not analyze the effects of individual doses of DEX and MPS separately, as none of our patients received MPS alone. Moreover, differences in corticosteroid bioavailability and metabolism, including half-life, duration of action, and frequency of administration, may have influenced our results. Future studies are essential to determine the threshold doses of each steroid treatment that increase the risk of ONFH. Finally, our retrospective study design prevents the establishment of a definitive causal relationship between cumulative steroid dose and ONFH severity. Furthermore, potential confounders that may influence ONFH development, such as preexisting vascular conditions, thrombophilic disorders, and genetic predispositions, were not fully accounted in this study. Therefore, future prospective cohort studies are essential to validate these findings and better clarify the role of these factors in ONFH progression.

## Conclusions

In summary, our study provides an insight into the relationship between COVID-19-related factors and the development of ONFH, emphasizing the critical role of cumulative steroid dose as the primary determinant of ONFH severity in post-COVID-19 patients. Additionally, our findings suggest that the combined use of corticosteroids may accelerate the onset of ONFH, underscoring the need for cautious management of steroid regimens in COVID-19 patients. While we found no significant associations between vaccination status, joint involvement, and COVID-19-related factors, these results underscore the complexity of ONFH pathogenesis. Future research is warranted to unravel the intricate mechanisms linking COVID-19, corticosteroid therapy, and ONFH development, which may ultimately guide more effective prevention and treatment strategies.

## Data Availability

The datasets used and/or analyzed during the current study are available from the corresponding author on reasonable request.

## Abbreviations

ACE2: Angiotensin converting enzyme 2
ARCO: Association Research Circulation Osseus
AVN: Avascular necrosis
BMI: Body mass index
COVID-19: Coronavirus disease 2019
CT: Computed tomography
DEX: Dexamethasone
FH: Femoral head
HHS: Harris hip score
HLA: Human leukocyte antigen
ICU: Intensive care unit
IL: Interleukin
IQR: Interquartile range
MPS: Methylprednisolone
MRI: Magnetic resonance imaging
ON: Osteonecrosis
ONFH: Osteonecrosis of the femoral head
PS: Prednisolone
RT-PCR: Real-time polymerase chain reaction
SARS: Severe acute respiratory syndrome
SARS-CoV-2: Severe acute respiratory syndrome coronavirus-2
SD: Standard deviation
THA: Total hip arthroplasty
THR: Total hip replacement
TNF: Tumor necrosis factor
VAS: Visual analog scale
vWF: von Willebrand factor
WHO: World Health Organization
