# Correction: Osteonecrosis of the femoral head in post-COVID-19 patients: a retrospective comparative study

**DOI:** 10.1186/s13018-025-06107-1

**Published:** 2025-08-21

**Authors:** Jichang Seong, Abduaziz Babakulov, Saodat Asilova, Babamukhamedova Shakhnoza, Makhmudova Nodira, Akbarjon Mirzayev

**Affiliations:** 1 School of Medicine, Central Asian University, 111221 Tashkent, Uzbekistan; 2 Department of Orthopedics and Traumatology, Akfa Medline University Hospital, 100211 Tashkent, Uzbekistan; 3 Department of Orthopedics and Traumatology, Kimyo University Hospital, 100121 Tashkent, Uzbekistan

**Correction: J Orthop Surg Res 20, 362 (2025)**

**https://doi.org/10.1186/s13018-025-05657-8**

Following the publication of the original article, the authors identified an error in Tables 1, 2, 3, 4, and 5. The corrected tables are given below and the changes have been highlighted in **bold typeface**.

In the abstract section, the percentage of COVID-19 lung involvement and the days of hospital stay in ICU should have been presented as median value instead of mean value.

The sentence currently reads:

 “The DEX and MPS-treated group had a greater extent of COVID-19 lung involvement compared to the DEX treated group (59.2% vs. 36.3%, p = 0.002), as well as longer hospital stays in both general ward (14.2 days vs. 10.6 days, p = 0.018) and ICU (5.4 days vs. 3 days, p = 0.017).”

The sentence should read:

“The DEX and MPS-treated group had a greater extent of COVID-19 lung involvement compared to the DEX treated group (**60% vs. 30%**, p = 0.002), as well as longer hospital stays in both general ward (14.2 days vs. 10.6 days, p = 0.018) and ICU (**5.5 days vs. 2 days, p = 0.020**).”

In the types of steroid regimen subsection of the result section, the percentage of COVID-19 lung involvement and the days of hospital stay in ICU should have been presented as median value instead of mean value.

The sentence currently reads:

 “However, patients treated with DEX and MPS had significantly greater COVID-19 lung involvement compared to those treated with DEX alone (59.2% vs. 36.3%, p = 0.002, d = 1.017). The DEX and MPS-treated group also had significantly longer hospitalization duration in both the general ward (14.2 days vs. 10.6 days, p = 0.018, d = 0.788) and ICU (5.4 days vs. 3 days, p = 0.017, d = 1.180).”

The sentence should read:

“However, patients treated with DEX and MPS had significantly greater COVID-19 lung involvement compared to those treated with DEX alone (**60% vs. 30%, p = 0.002, r**_**B**_** = 0.565**). The DEX and MPS-treated group also had significantly longer hospitalization duration in both the general ward (14.2 days vs. 10.6 days, p = 0.018, d = 0.788) and ICU (**5.5 days vs. 2 days, p = 0.020, r**_**B**_** = 0.620**).”

In the 4th paragraph of the discussion section, one of the abbreviations was misspelled.

The sentence currently reads:

“In the context of COVID-19, a case report described the use of combined DEX and MP, with the ONFH symptoms developing 56 days after recovery.”

The sentence should read:

“In the context of COVID-19, a case report described the use of combined DEX and **MPS**, with the ONFH symptoms developing 56 days after recovery.”

In the author contributions section, one of the words was misspelled.

The sentence currently reads:

“Fromal Analysis, J.S. and A.M.”

The sentence should read:

“**Formal** Analysis, J.S. and A.M.”

Incorrect Table 1:


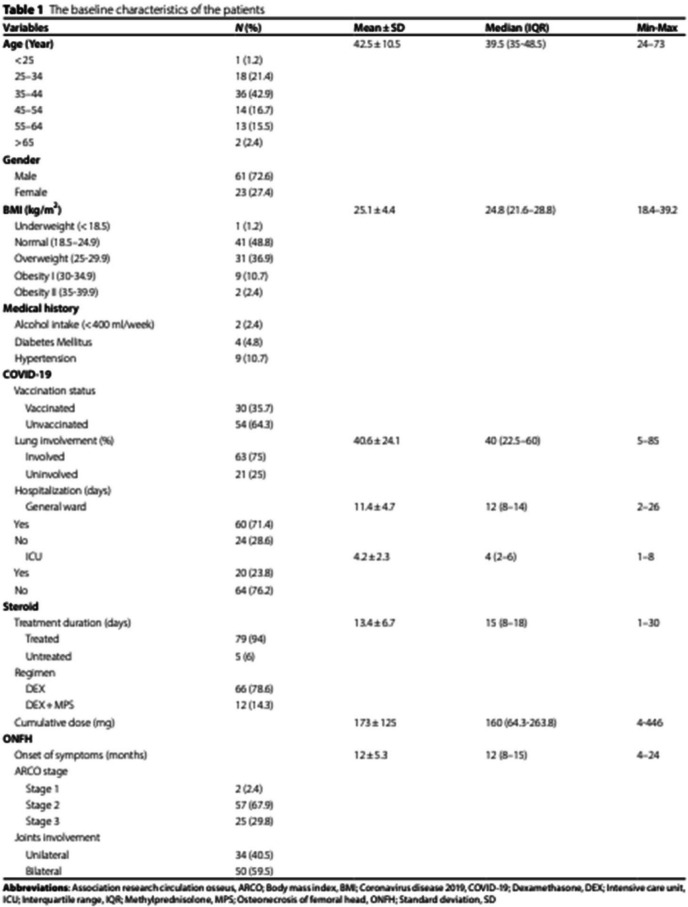


Correct

**Table 1:** The baseline characteristics of the patients

Variables	N (%)	Mean ± SD	Median (IQR)	Min–Max
**Age (Year)**		42.5 ± 10.5	39.5 (35–48.5)	24–73
< 25	1 (1.2)			
25–34	18 (21.4)			
35–44	36 (42.9)			
45–54	14 (16.7)			
55–64	13 (15.5)			
> 65	2 (2.4)			
**Gender**				
Male	61 (72.6)			
Female	23 (27.4)			
**BMI (kg/m**^**2**^**)**		25.1 ± 4.4	24.8 (21.6–28.8)	18.4–39.2
Underweight (< 18.5)	1 (1.2)			
Normal (18.5–24.9)	41 (48.8)			
Overweight (25–29.9)	31 (36.9)			
Obesity I (30–34.9)	9 (10.7)			
Obesity II (35–39.9)	2 (2.4)			
**Medical history**				
Alcohol intake (< 400 ml/week)	2 (2.4)			
Diabetes Mellitus	4 (4.8)			
Hypertension	9 (10.7)			
**COVID-19**				
Vaccination status				
Vaccinated	30 (35.7)			
Unvaccinated	54 (64.3)			
Lung involvement (%)		40.6 ± 24.1	40 (22.5–60)	5–85
Involved	63 (75)			
Uninvolved	21 (25)			
Hospitalization (days)				
General ward		11.4 ± 4.7	12 (8–14)	2–26
Yes	60 (71.4)			
No	24 (28.6)			
ICU		4.2 ± 2.3	4 (2–6)	1–8
Yes	20 (23.8)			
No	64 (76.2)			
**Steroid**				
Treatment duration (days)		13.4 ± 6.7	15 (8–18)	1–30
Treated	**78 (92.9)**			
Untreated	**6 (7.1)**			
Regimen				
DEX	66 (78.6)			
DEX + MPS	12 (14.3)			
Cumulative dose (mg)		173 ± 125	160 (64.3–263.8)	4–446
**ONFH**				
Symptom onset (months)		12 ± 5.3	12 (8–15)	4–24
ARCO stage				
Stage 1	2 (2.4)			
Stage 2	57 (67.9)			
Stage 3	25 (29.8)			
Joints involvement				
Unilateral	34 (40.5)			
Bilateral	50 (59.5)			

*Abbreviations*: *ARCO* Association research circulation osseus, *BMI* Body mass index, *COVID-19* Coronavirus disease 2019, *DEX* Dexamethasone, *ICU* Intensive care unit, *IQR* Interquartile range, *MPS* Methylprednisolone, *ONFH* Osteonecrosis of femoral head, *SD* Standard deviation

Incorrect Table 2:


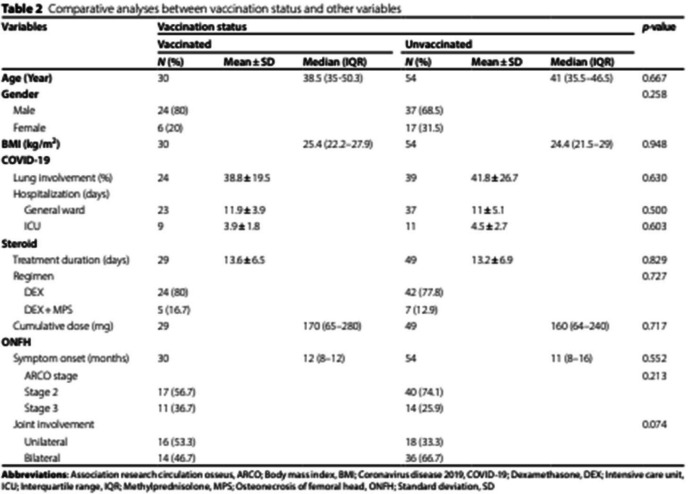


Correct

**Table 2:** Comparative analyses between vaccination status and other variables

Variables	Vaccination status						*p*-value
	**Vaccinated**			**Unvaccinated**			
	**N (%)**	**Mean ± SD**	**Median (IQR)**	**N (%)**	**Mean ± SD**	**Median (IQR)**	
**Age (Year)**	30		38.5 (35–50.3)	54		41 (35.5–46.5)	0.667
**Gender**							0.258
Male	24 (80)			37 (68.5)			
Female	6 (20)			17 (31.5)			
**BMI (kg/m**^**2**^**)**	30		25.4 (22.2–27.9)	54		24.4 (21.5–29)	0.948
**COVID-19**							
Lung involvement (%)	24		**35 (25–50)**	39		**40 (17.5–60)**	**0.804**
Hospitalization (days)							
General ward	23	11.9 ± 3.9		37	11 ± 5.1		0.500
ICU	9	3.9 ± 1.8		11	4.5 ± 2.7		0.603
**Steroid**							
Treatment duration (days)	29		**15 (8–18)**	49		**14 (8–18)**	**0.791**
Regimen							0.727
DEX	24 (80)			42 (77.8)			
DEX + MPS	5 (16.7)			**7 (13)**			
Cumulative dose (mg)	29		170 (65–280)	49		160 (64–240)	0.717
**ONFH**							
Symptom onset (months)	30		12 (8–12)	54		11 (8–16)	0.552
ARCO stage							0.213
Stage 2	17 (56.7)			40 (74.1)			
Stage 3	11 (36.7)			14 (25.9)			
Joint involvement							0.074
Unilateral	16 (53.3)			18 (33.3)			
Bilateral	14 (46.7)			36 (66.7)			

*Abbreviations*: *ARCO* Association research circulation osseus, *BMI* Body mass index, *COVID-19* Coronavirus disease 2019, *DEX* Dexamethasone, *ICU* Intensive care unit, *IQR* Interquartile range, *MPS* Methylprednisolone, *ONFH* Osteonecrosis of femoral head, *SD* Standard deviation

Incorrect Table 3:


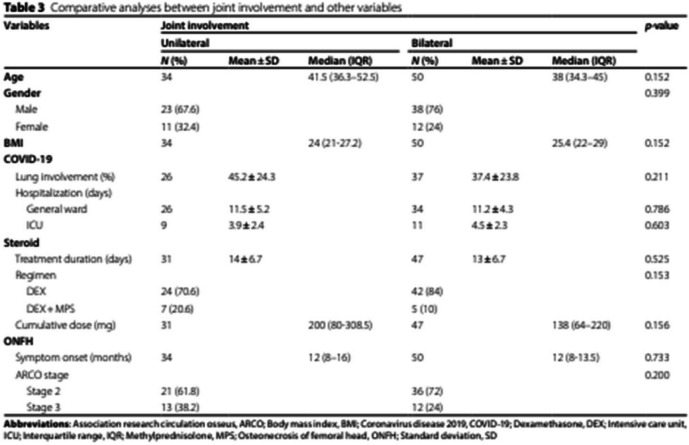


Correct

**Table 3:** Comparative analyses between joint involvement and other variables

Variables	Joint involvement						*p*-value
	**Unilateral**			**Bilateral**			
	**N (%)**	**Mean ± SD**	**Median (IQR)**	**N (%)**	**Mean ± SD**	**Median (IQR)**	
**Age**	34		41.5 (36.3–52.5)	50		38 (34.3–45)	0.152
**Gender**							0.399
Male	23 (67.6)			38 (76)			
Female	11 (32.4)			12 (24)			
**BMI**	34	**24.3 ± 4.2**		50	**25.7 ± 4.5**		**0.156**
**COVID-19**							
Lung involvement (%)	26		**42.5 (26.3–65)**	37		**30 (20–60)**	**0.210**
Hospitalization (days)							
General ward	26	11.5 ± 5.2		34	11.2 ± 4.3		0.786
ICU	9	3.9 ± 2.4		11	4.5 ± 2.3		0.603
**Steroid**							
Treatment duration (days)	31	14 ± 6.7		47	13 ± 6.7		0.525
Regimen							0.153
DEX	24 (70.6)			42 (84)			
DEX + MPS	7 (20.6)			5 (10)			
Cumulative dose (mg)	31		200 (80–308.5)	47		138 (64–220)	0.156
**ONFH**							
Symptom onset (months)	34		12 (8–16)	50		12 (8–13.5)	0.733
ARCO stage							0.200
Stage 2	21 (61.8)			36 (72)			
Stage 3	13 (38.2)			12 (24)			

*Abbreviations*: *ARCO* Association research circulation osseus, *BMI* Body mass index, *COVID-19* Coronavirus disease 2019, *DEX* Dexamethasone, *ICU* Intensive care unit, *IQR* Interquartile range, *MPS* Methylprednisolone, *ONFH* Osteonecrosis of femoral head, *SD* Standard deviation

Incorrect Table 4:


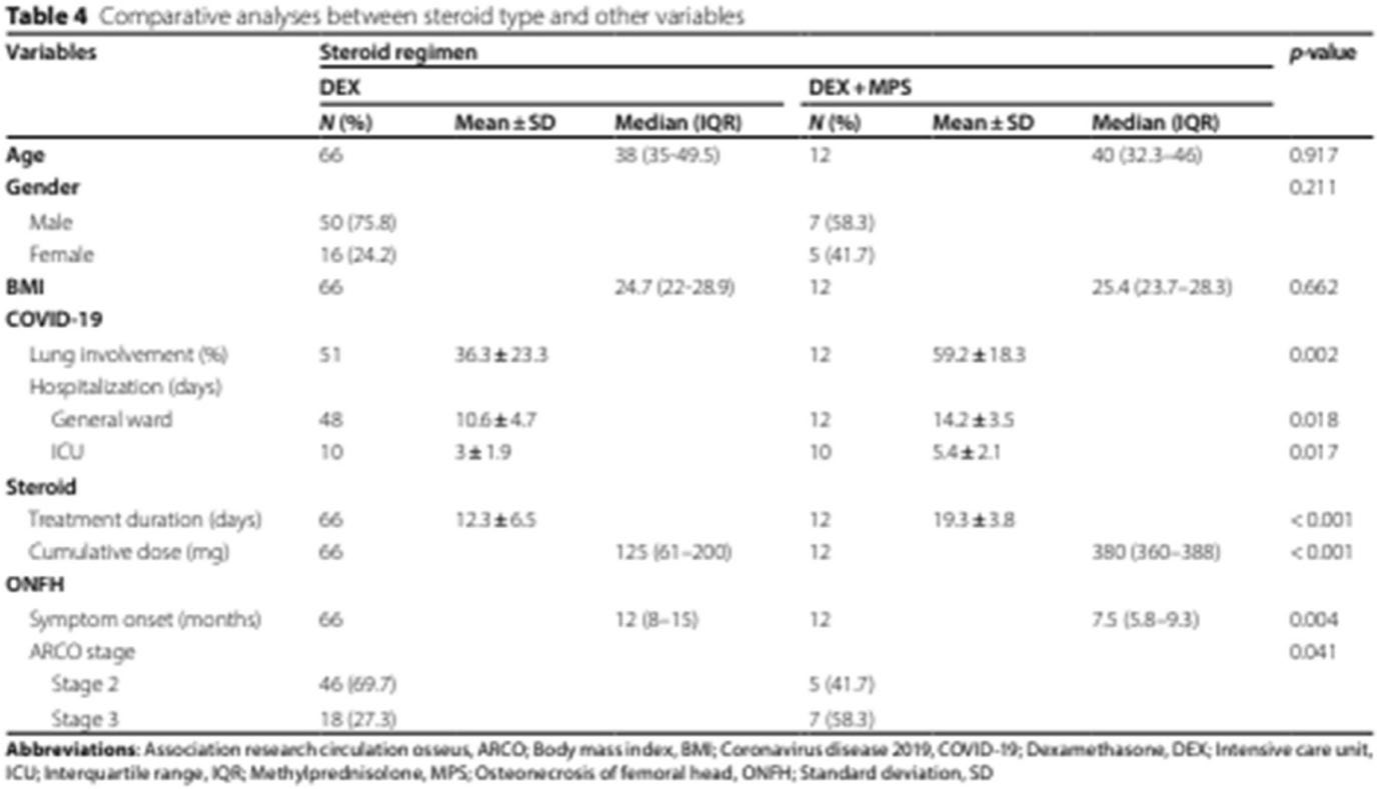


Correct

**Table 4:** Comparative analyses between steroid type and other variables

Variables	Steroid regimen						*p*-value
	**DEX**			**DEX + MPS**			
	**N (%)**	**Mean ± SD**	**Median (IQR)**	**N (%)**	**Mean ± SD**	**Median (IQR)**	
**Age**	66		38 (35–49.5)	12		40 (32.3–46)	0.917
**Gender**							0.211
Male	50 (75.8)			7 (58.3)			
Female	16 (24.2)			5 (41.7)			
**BMI**	66		24.7 (22–28.9)	12		25.4 (23.7–28.3)	0.662
**COVID-19**							
Lung involvement (%)	51		**30 (20–50)**	12		**60 (43.8–76.3)**	0.002
Hospitalization (days)							
General ward	48	10.6** ± **4.7		12	14.2** ± **3.5		0.018
ICU	10		**2 (2–4.5)**	10		**5.5 (4–7)**	**0.020**
**Steroid**							
Treatment duration (days)	66	12.3** ± **6.5		12	19.3** ± **3.8		< 0.001
Cumulative dose (mg)	66		125 (61–200)	12		380 (360–388)	< 0.001
**ONFH**							
Symptom onset (months)	66		12 (8–15)	12		7.5 (5.8–9.3)	0.004
ARCO stage							0.041
Stage 2	46 (69.7)			5 (41.7)			
Stage 3	18 (27.3)			7 (58.3)			

*Abbreviations*: *ARCO* Association research circulation osseus, *BMI* Body mass index, *COVID-19* Coronavirus disease 2019, *DEX* Dexamethasone, *ICU* Intensive care unit, *IQR* Interquartile range, *MPS* Methylprednisolone, *ONFH* Osteonecrosis of femoral head, *SD* Standard deviation

Incorrect Table 5:


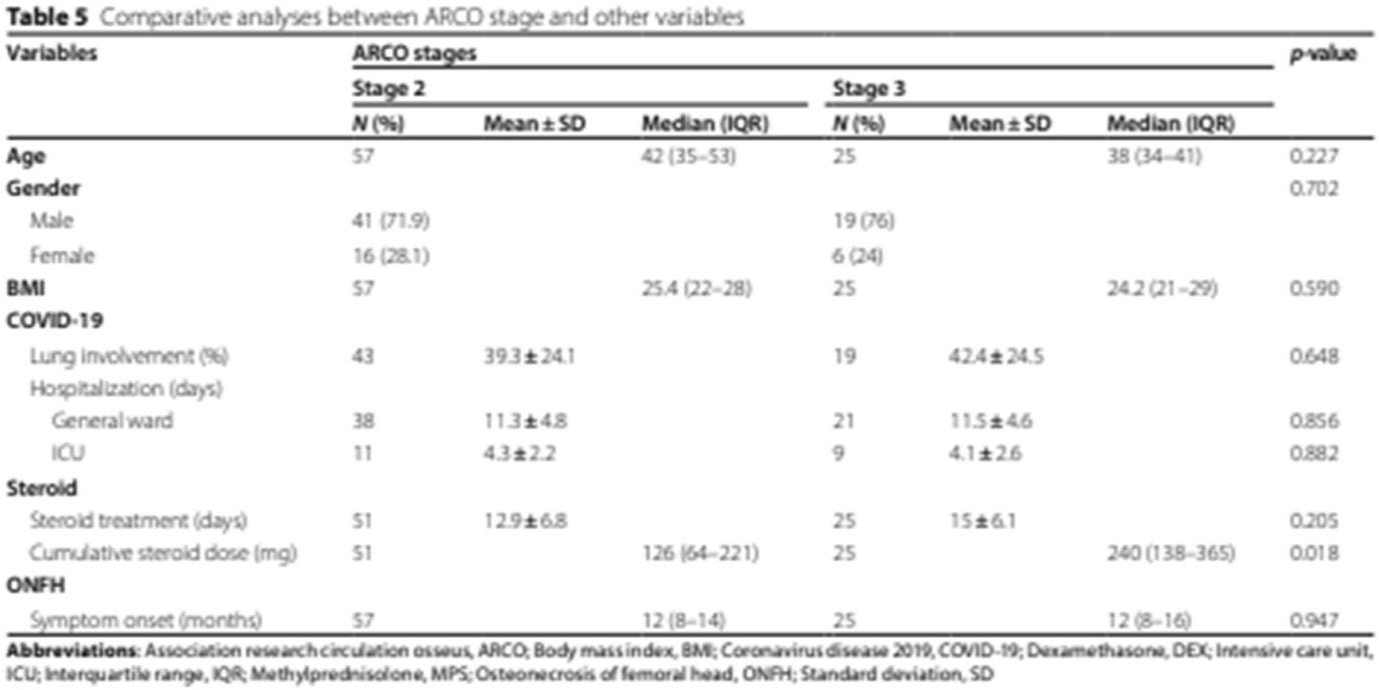


Correct

**Table 5:** Comparative analyses between ARCO stage and other variables

Variables	ARCO stages						*p*-value
	**Stage 2**			**Stage 3**			
	**N (%)**	**Mean ± SD**	**Median (IQR)**	**N (%)**	**Mean ± SD**	**Median (IQR)**	
**Age**	57		42 (35–53)	25		38 (34–41)	**0.104**
**Gender**							0.702
Male	41 (71.9)			19 (76)			
Female	16 (28.1)			6 (24)			
**BMI**	57	**25.3 ± 4.5**		25	**24.8 ± 4.4**		**0.600**
**COVID-19**							
Lung involvement (%)	43		**35 (22.5–55)**	19		**40 (22.5–55)**	**0.657**
Hospitalization (days)							
General ward	38	11.3 ± 4.8		21	11.5 ± 4.6		0.856
ICU	11		**5 (2.5–6)**	9		**3 (2–6)**	**0.939**
**Steroid**							
Treatment duration (days)	51	12.9 ± 6.8		25	15 ± 6.1		0.205
Cumulative dose (mg)	51		126 (64–221)	25		240 (138–365)	0.018
**ONFH**							
Symptom onset (months)	57		12 (8–14)	25		12 (8–16)	0.947

*Abbreviations*: *ARCO* Association research circulation osseus, *BMI* Body mass index, *COVID-19* Coronavirus disease 2019, *DEX* Dexamethasone, *ICU* Intensive care unit, *IQR* Interquartile range, *MPS* Methylprednisolone, *ONFH* Osteonecrosis of femoral head, *SD* Standard deviation

The authors apologize for these errors. The original article has been corrected.

